# Osthole Suppresses Knee Osteoarthritis Development by Enhancing Autophagy Activated via the AMPK/ULK1 Pathway

**DOI:** 10.3390/molecules27238624

**Published:** 2022-12-06

**Authors:** Teng Ma, Xiangpeng Wang, Wenjing Qu, Lingsen Yang, Cheng Jing, Bingrui Zhu, Yongkui Zhang, Wenpeng Xie

**Affiliations:** 1 First Clinical Medical College, Shandong University of Traditional Chinese Medicine, Jinan 250355, China; 2 Department of Orthopedics, Affiliated Hospital of Shandong University of Traditional Chinese Medicine, Jinan 250011, China

**Keywords:** osthole, AMPK/ULK1, autophagy, degeneration, knee osteoarthritis

## Abstract

Knee osteoarthritis (KOA) is an increasingly prevalent heterogeneous disease characterized by cartilage erosion and inflammation. As the main chemical constituent of Angelicae Pubescentis Radix (APR), an anti-inflammatory herbal medicine, the potential biological effects and underlying mechanism of osthole on chondrocytes and KOA progression remain elusive. In this study, the potential effect and mechanism of osthole on KOA were investigated in vitro and in vivo. We found that osthole inhibited IL-1β-induced apoptosis and cartilage matrix degeneration by activating autophagy in rat chondrocytes. In addition, osthole could activate autophagy through phosphorylation of AMPK/ULK1, and AMPK serves as a positive upstream regulator of ULK1. Furthermore, KOA rats treated with osthole showed phosphorylation of the AMPK/ULK1 pathway and autophagy activation, as well as cartilage protection. Collectively, the AMPK/ULK1 signaling pathway can be activated by osthole to enhance autophagy, thereby suppressing KOA development. Osthole may be a novel and effective therapeutic agent for the clinical treatment of KOA.

## 1. Introduction

Knee Osteoarthritis (KOA), as a heterogeneous disease with complex etiology, is characterized by joint pain, deformity, and dysfunction [1]. Reported studies have shown that abnormal cartilage matrix metabolism and chondrocyte apoptosis, two major factors in the occurrence of osteoarthritis (OA), are closely related to chondrocyte autophagy [2,3]. In view of the high incidence of KOA and the imperfection of nonsurgical treatments such as nonsteroidal anti-inflammatory drugs (NSAIDs) [4], other complementary and alternative medicines, including traditional Chinese medicine (TCM), have become increasingly utilized.

Osthole (7-Methoxy-8-isopentenylcoumarin), the main ingredient of herbal medicine Angelicae pubescentis Radix (APR, the root of Freziera biserrate, also known as Angelica pubescens Maxim, and Duhuo), can inhibit reactive oxygen species (ROS) production and stimulate bone formation [5,6,7]. Furthermore, osthole has been shown to reduce cartilage degeneration and thereby suppress KOA development, but its protective mechanism remains unclear [8]. As a widely used herbal medicine for dispelling wind, removing dampness, and relieving pain, APR has been applied in East Asian countries for thousands of years [9]. Our previous studies have shown that the Cangxitongbi (CXTB) capsule, a Chinese patent medicine mainly composed of APR, can protect articular cartilage by enhancing autophagy, suppressing inflammation, and inhibiting cartilage extra-cellular matrix (ECM) degradation [10,11]. Given the high content of osthole in CXTB, it is necessary to further verify the mechanism of osthole in inhibiting KOA cartilage degeneration.

The occurrence and development of KOA is thought to be related to the imbalance of AMP-activated kinase (AMPK), as attenuated phosphorylation of AMPKα1 is reported in the cartilage of KOA mice [12]. AMPK, as an energy regulator that plays a role in cell metabolic balance, has been proven to be involved in apoptosis and autophagy [13]. Phosphorylated AMPK can both inhibit and promote autophagy [3,14]. Autophagy is considered to be essential for maintaining homeostasis in eukaryotic cells, and chondrocytes in the early stage of OA are protected by autophagy [15,16]. Given the role of CXTB capsule as well as osthole in the treatment of KOA, we hypothesized that osthole may activate autophagy to exert its anti-KOA effect by targeting the AMPK/ULK1 pathway [10].

In this study, we investigated the therapeutic effect of osthole on chondrocytes degeneration induced by IL-1β and knee cartilage tissue of KOA rat models. In vitro, we then determined the contribution of autophagy to this effect by using 3-methyladenine (3-MA) and evaluated osthole-mediated autophagy activation via the AMPK/ULK1 pathway using Dorsomorphin.

## 2. Results

### 2.1. Osthole Rescues Chondrocytes Treated with IL-1β

**Figure 1:**
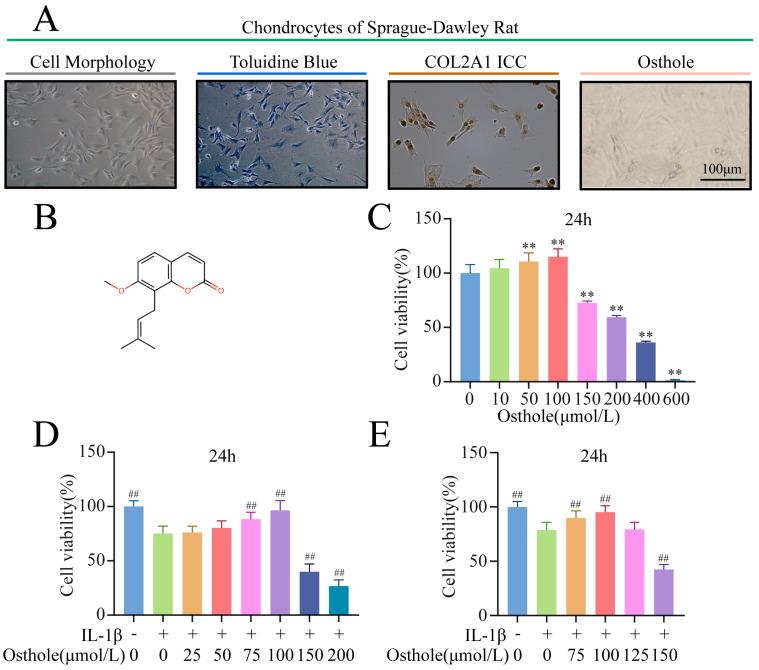
The effect of osthole on the viability of chondrocytes. (**A**) Verification of chondrocytes with toluidine blue and IHC for COL2A1. Morphology of chondrocytes treated with 100 μM osthole. (**B**) Chemical structure of osthole. (**C**) CCK−8 results of chondrocytes treated with different concentrations of osthole for 24 h. (**D**,**E**) CCK−8 results of osthole−pretreated chondrocytes induced by IL−1β for 24 h. All data were presented as mean ± SD (*n* = 3). ** *p* < 0.01 compared to the control group. ## *p* < 0.01 compared to the IL−1β group. IHC, Immunohistochemistry; CCK−8, Cell Counting Kit−8; IL−1β, interleukin−1β.

Most chondrocytes that grew adherent to the wall were polygonal in shape. Their nuclei were blue after toluidine blue staining and dark brown after COL2A1 immunocytochemical staining. The morphology of chondrocytes treated with 100 μM osthole was not altered (Figure 1A). In the CCK-8 assay, cell viability decreased significantly when the concentration of osthole exceeded 150 µM, while concentrations ≤ 100 µM were non-toxic to chondrocytes at 24 h (Figure 1C). A concentration of 100 µM osthole showed the strongest protective effects against IL-1β (10 ng/mL) (Figure 1D,E). These results demonstrated that the optimal dose of osthole for chondrocyte rescue was 100 µM.

### 2.2. Osthole Delays OA-Related Degeneration in IL-1β-Treated Chondrocytes

**Figure 2:**
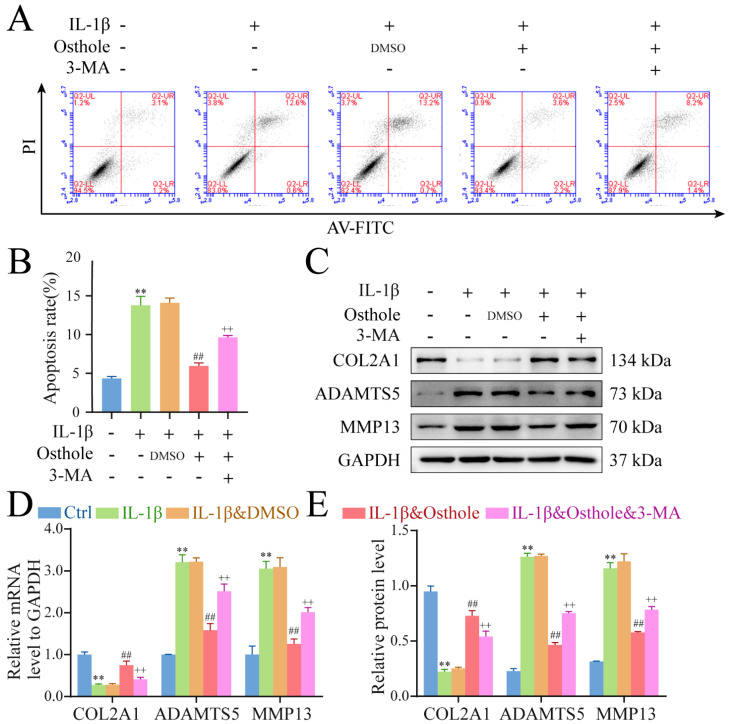
Osthole delays OA−related degeneration in IL−1β−treated chondrocytes. (**A**,**B**) Flow cytometry results of chondrocytes treated with osthole and IL−1β were analyzed. (**C**−**E**) Western blot and qPCR analysis of COL2A1, ADAMTS5, and MMP13. All data were presented as mean ± SD (*n* = 3). ** *p* < 0.01 compared to the control group. ## *p* < 0.01 compared to the IL−1β group. ++ *p* < 0.01 compared to the osthole group. OA, osteoarthritis; IL−1β, interleukin−1β; qPCR, quantitative real−time polymerase chain reaction; COL2A1, Collagen type II α1; ADAMTS5, a disintegrin and metalloproteinase with thrombospondin motifs−5; MMP13, matrix metalloprotein−13.

Annexin V/PI was used to detect the apoptosis of chondrocytes in each group. As shown in Figure 2A,B, IL-1β could induce chondrocyte apoptosis (13.77 ± 1.16%), whereas 0.1% DMSO had no effect on chondrocyte apoptosis (14.10 ± 0.62%). However, with the addition of 100 µM osthole, the percentage of cells undergoing IL-1β-induced apoptosis dropped to 5.97 ± 0.38%. Assessment of the anabolism marker (COL2A1) and catabolism markers (ADAMTS5 and MMP13) with Western Blot and qPCR showed higher protein and mRNA levels of COL2A1 and decreases in ADAMTS5 and MMP13 in osthole-treated chondrocytes (Figure 2C–E) relative to models. These results suggested that osthole inhibited apoptosis and cartilage matrix degradation.

### 2.3. Osthole Attenuates IL-1β-Induced Chondrocyte Degeneration by Activating Autophagy

**Figure 3:**
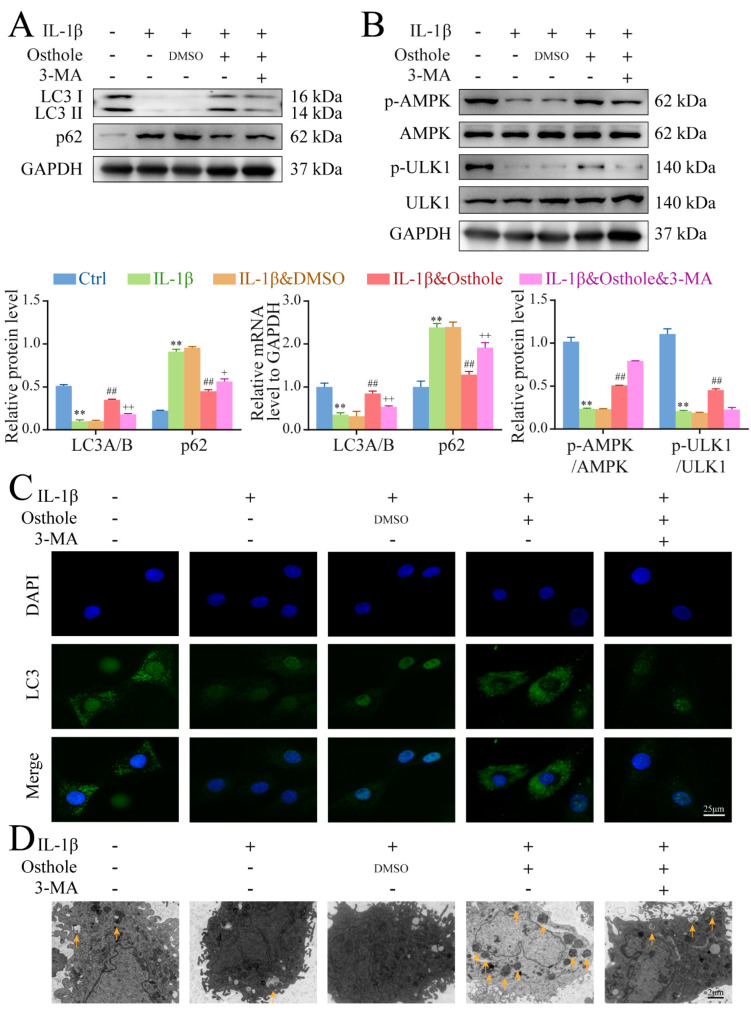
Osthole activates autophagy and the AMPK/ULK1 signaling pathway in IL−1β−treated rat chondrocytes. (**A**) Western blot and qPCR analysis of LC3 II and p62 levels in chondrocytes. (**B**) Western blot analysis of p−AMPK, AMPK, p−ULK1, and ULK1 protein expression in chondrocytes. (**C**) The LC3 expression was detected by the immunofluorescence. (**D**) Autophagosomes were observed using TEM. The yellow arrows in the TEM images represent autophagosomes. Data represent mean ± SD (*n* = 3). ** *p* < 0.01 compared to the control group. ## *p* < 0.01 compared to the IL−1β group. + *p* < 0.05, ++ *p* < 0.01 compared to the osthole group. IL−1β, interleukin−1β; qPCR, quantitative real−time polymerase chain reaction; AMPK, 5′ AMP−activated protein kinase; ULK1, Unc−51−like kinase 1; TEM, transmission electron microscopy.

To investigate the activation effect of osthole on autophagy, we evaluated the protein and mRNA levels of autophagy marker (LC3 II/I and p62) and observed the number of autophagosomes (APs) in chondrocytes of each group by TEM. In addition, autophagy inhibitor 3-MA was used to incubate IL-1β-treated chondrocytes to demonstrate the role of autophagy in osthole-mediated attenuation of OA-related degeneration. The expression of LC3 II protein and mRNA in chondrocytes treated with IL-1β for 24 h was decreased, whereas the expression of p62 was increased relative to controls, which was consistent with a previous report [17]. Similarly, immunofluorescence staining data showed that the level of LC3 II in IL-1β-treated chondrocytes was significantly reduced, as was the number of APs. We also found that the expression of p-AMPK and p-ULK1 was obviously downregulated in IL-1β-treated chondrocytes. However, after the addition of osthole, the expression of p62 decreased, while LC3 II/I, p-AMPK, and p-ULK1 increased (Figure 3A,B). Immunofluorescence staining data and TEM results showed that osthole restored the expression of LC3 II and number of Aps (Figure 3C,D). These effects were reversed by 3-MA, indicating that osthole could attenuate IL-1β-induced chondrocyte degeneration and apoptosis by activating autophagy.

### 2.4. Autophagy Is Promoted by Phosphorylating AMPK/ULK1 in Osthole-Treated Chondrocytes

**Figure 4:**
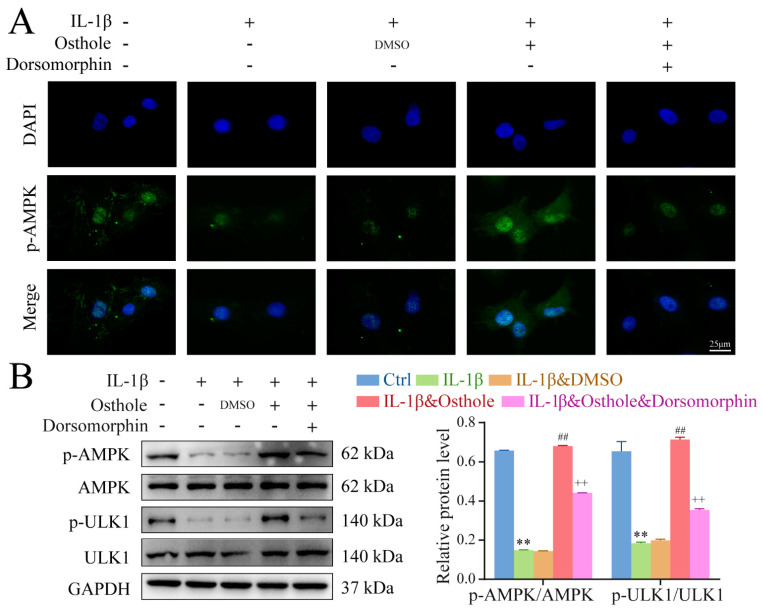
Osthole phosphorylates AMPK/ULK1 in IL−1β−treated rat chondrocytes. (**A**) The representative p−AMPK expression was detected by the immunofluorescence. (**B**) Western blot analysis of p−AMPK, AMPK, p−ULK1, and ULK1 protein expression in chondrocytes. Data represent mean ± SD (*n* = 3). ** *p* < 0.01 compared to the control group. ## *p* < 0.01 compared to the IL−1β group. ++ *p* < 0.01 compared to the osthole group. IL−1β, interleukin−1β; AMPK, 5′ AMP−activated protein kinase; ULK1, Unc−51−like kinase 1.

**Figure 5:**
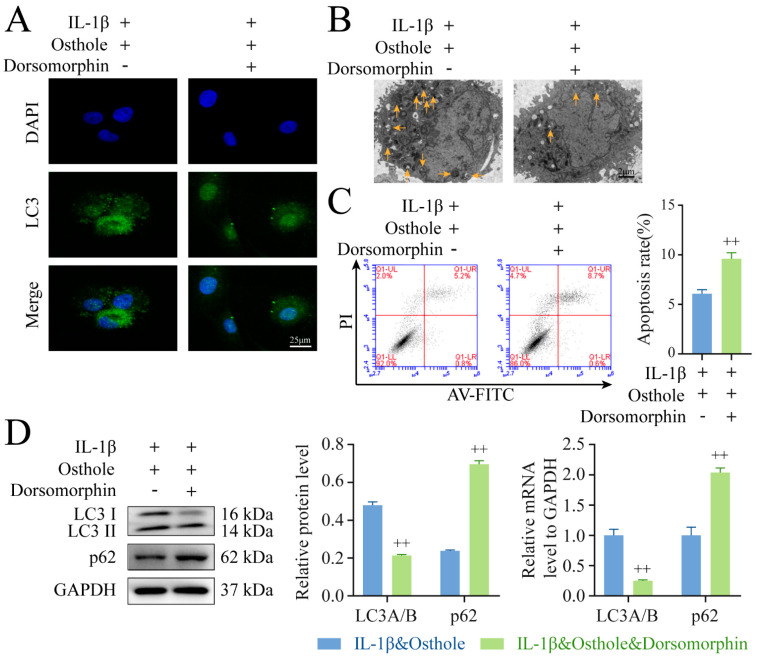
Autophagy is promoted by phosphorylating AMPK/ULK1 in osthole−treated chondrocytes. (**A**) The representative LC3 expression was detected by the immunofluorescence. (**B**) Autophagosomes were observed using TEM. The yellow arrows in the TEM images represent autophagosomes. (**C**) Flow cytometry results of chondrocytes treated with or without Dorsomorphin were analyzed. (**D**) Western blot and qPCR analysis of LC3 II and p62 levels in chondrocytes. Data represent mean ± SD (*n* = 3). ++ *p* < 0.01 compared to the osthole group. IL−1β, interleukin−1β; AMPK, 5′ AMP−activated protein kinase; ULK1, Unc−51−like kinase 1; TEM, transmission electron microscopy; qPCR, quantitative real−time polymerase chain reaction.

Given the high expression of p-AMPK and p-ULK1 following treatment of chondrocytes with osthole, we next sought to determine whether AMPK/ULK1 was involved in the induction of autophagy. Immunofluorescence staining, TEM, immunoblot, and qPCR were employed to evaluate autophagy induction in the presence of the AMPK inhibitor Dorsomorphin. Treatment with Dorsomorphin reversed the observed upregulation of p-AMPK, p-ULK1, and LC3 II/I levels, and the down-regulation of p62 by osthole in chondrocytes, as well as the high number of Aps (Figure 4 and Figure 5). Additionally, Dorsomorphin treatment reversed the decrease of apoptosis percentages in IL-1β-treated chondrocytes by osthole. These results demonstrated that osthole inhibits chondrocytes apoptosis by activating autophagy via phosphorylating AMPK/ULK1.

### 2.5. Osthole Suppresses KOA Progression in a Dose-Dependent Manner In Vivo

**Figure 6:**
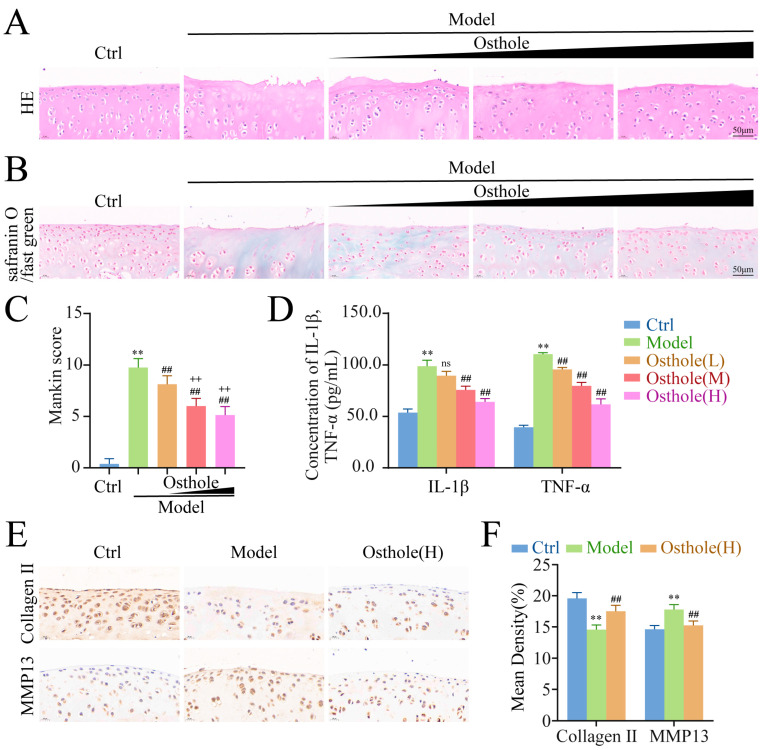
Osthole attenuates cartilage injury and suppresses inflammation in a dose-dependent manner. (**A**–**C**) HE staining, safranin O/fast green staining, and Mankin score of groups (*n* = 8). (**D**) IL-1β and TNF-α levels in serum measured by ELISA. (**E**,**F**) Immunohistochemical staining of Collagen II and MMP13 in each group (*n* = 8). ** *p* < 0.01 compared to the control group. ## *p* < 0.01 compared to the model group. ++ *p* < 0.01 compared to the osthole low-dose group. And ns *p* > 0.05 compared to the model group. HE, Hematoxylin and eosin; IL-1β, interleukin-1β; TNF-α, tumor necrosis factor-α; ELISA, enzyme-linked immunosorbent assay.

The hematoxylin and eosin (HE) and safranin O/fast green images and Mankin scores of articular tissues of each group are shown in Figure 6A,B. Compared with the control group, the articular cartilage surface of rats treated using the modified Hulth method was rough, the arrangement of cells was disordered, and the Mankin score was higher, which indicated that the modeling was successful. After treatment with osthole, the cartilage of the knee joint of rats was preserved, and the chondrocyte arrangement was relatively in order. Moreover, the Mankin scores of the low-, mid-, and high-dose osthole groups were gradually lower than those of the model group. The results of IHC indicated that the level of collagen II was lower and the level of MMP13 was higher in the model group compared with the control group, and the expression of collagen II was rescued and the level of MMP13 was decreased after high-dose osthole treatment (Figure 6E,F). The above data suggested that osthole exerted a protective effect on articular cartilage.

In line with the trend of Mankin scores, the contents of IL-1β and TNF-α in serum were decreased in a dose-dependent manner by three doses of osthole (Figure 6C,D). This suggested that osthole could suppress inflammation in a dose-dependent manner by reducing the levels of IL-1β and TNF-α.

### 2.6. High Dose Osthole Phosphorylates AMPK/ULK1 and Activates Autophagy In Vivo

**Figure 7:**
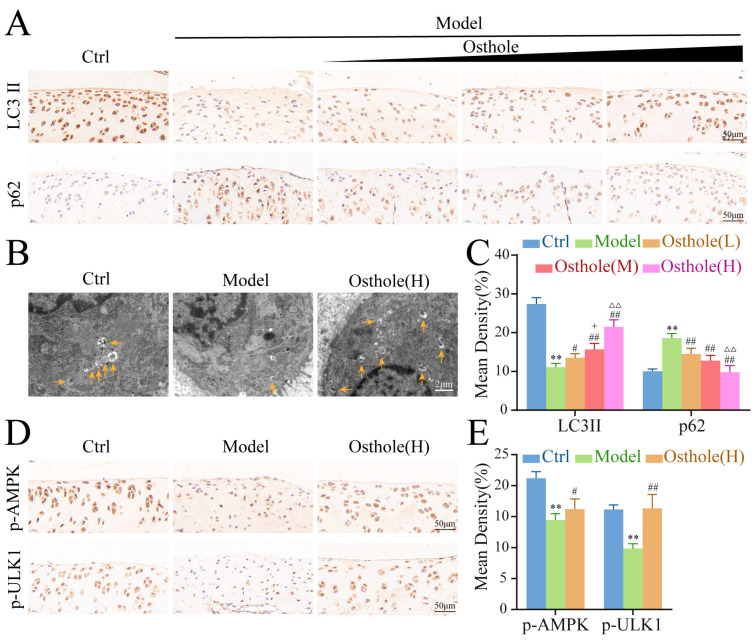
High dose osthole phosphorylates AMPK/ULK1 and activates autophagy in vivo. (**A**,**C**) Immunohistochemical staining of LC3 II and p62 in each group (*n* = 8). (**B**) TEM images of the cartilage tissue. The yellow arrows in the TEM images represent autophagosomes. (**D**,**E**) Immunohistochemical staining of p-AMPK and p-ULK1 in each group (*n* = 8). ** *p* < 0.01 compared to the control group. # *p* < 0.05, ## *p* < 0.01 compared to the model group. + *p* < 0.05 compared to the osthole low-dose group. ΔΔ *p* < 0.01 compared to the osthole mid-dose group. AMPK, 5′ AMP-activated protein kinase; ULK1, Unc-51-like kinase 1; TEM, transmission electron microscopy.

To further explore the protective mechanism of osthole in articular cartilage, the levels of LC3 II/I, p62, p-AMPK, and p-ULK1, along with the number of APs in cartilage tissue, were detected by IHC and TEM. The results showed that the levels of LC3 II and p62 were dose-dependently elevated and decreased, respectively, in the articular cartilage of the osthole group compared to the KOA group (Figure 7A,B). Furthermore, TEM of cartilage tissue revealed that there were fewer autophagic vacuoles with double membrane structures in the KOA rats than in the controls, but more autophagic vacuoles could be observed after high-dose osthole treatment (Figure 7C), suggesting that administration of osthole dose-dependently activated autophagy in the KOA rat model.

The results of IHC indicated that the phosphorylation levels of AMPK and ULK1 in model group were lower compared with the control group, and the expression of p-AMPK and p-ULK1 was rescued after high-dose osthole treatment (Figure 7D,E). These results indicated that high doses of osthole significantly phosphorylated AMPK/ULK1 in vivo.

## 3. Discussion

The pathogenesis and phenotype of KOA are still being explored, and presently available treatments have unsatisfactory therapeutic efficacy. In view of the clinical efficacy and low cost of TCM in treatment of KOA, the effect of the commonly used herbal extracts merits further study [9]. In this study, in vitro experiments indicated that osthole regulated the AMPK/ULK1 pathway and autophagy, thus restraining apoptosis and metabolic abnormalities in IL-1β-induced chondrocytes. Moreover, in vivo experiments suggested that osthole administered by gavage improved the structure of the injured cartilage and activated autophagy and the AMPK/ULK1 pathway in a KOA rat model.

IL-1β is a pro-inflammatory factor related to OA, exposure of cultured chondrocytes to which is a classic in vitro model of OA [18]. IL-1β has been shown to impair cartilage matrix anabolism while inducing chondrocyte apoptosis and cartilage-matrix catabolism, which is consistent with the results of this study [19]. Similarly, 10 ng/mL IL-1β has been reported to reduce the expression of autophagy-related proteins in chondrocytes, which we have here recapitulated. Cartilage matrix degradation is accompanied by the development of KOA. The degradation of Collagen II and Aggrecan in the articular cartilage ECM is mediated by enzymes in the matrix metalloproteinase (MMP) and a disintegrin and metalloproteinase with thrombospondin motifs (ADAMTS) families, among which, MMP13 and AMAMTS5 are considered to play the most important roles [20,21]. In our study, osthole effectively reduced the protein and transcriptional levels of MMP13 and ADAMTS5 while upregulating the expression of COL2A1. It can thus be inferred that osthole can protect the cartilage matrix from degradation.

Additionally, the apoptosis rate of chondrocytes treated with osthole was lower than that of OA chondrocytes by flow cytometry, which indicated that osthole also had an anti-apoptotic effect. Osthole has been shown to slow down apoptosis in cardiomyocytes, and the results of TUNEL assay, Hoechst staining, and flow cytometry suggest that the degree of apoptosis in the osthole group was significantly lower than that in the model group [22]. Similarly, osthole exerted anti-apoptotic effects in the treatment of Alzheimer’s disease. Osthole-treated SH-SY5Y cells showed significantly lower TUNEL positivity than the model group, and osthole inhibited the expression of Bax and Caspase-3 in the apoptotic SH-SY5Y cells [23]. These studies support our conclusions and fully demonstrate the inhibitory effect of osthole on apoptosis. The role of osthole in vivo was also explored using a KOA rat model. Histological analysis by HE and safranin O/fast green staining, and quantitative analysis by Mankin scores showed that treatment with osthole inhibited the progression of KOA, as evidenced by lessened cartilage erosion and lower Mankin scores compared with untreated or mock-treated KOA rats. IL-1β and TNF-α, which can suppress ECM synthesis and promote apoptosis, are considered to be representative pro-inflammatory factors and catabolic markers of OA [24]. Previous studies have confirmed that IL-1β and TNF-α can be used as upstream targets of MMP13 and ADAMTS5 to induce OA [25,26]. Therefore, the inhibitory effect of osthole on IL-1β and TNF-α was examined in this study, which further revealed the anti-KOA function of osthole.

Autophagy digests damaged organelles and misfolded or aberrantly expressed proteins through the lysosomal pathway, thus maintaining the stability of the intracellular environment and playing an active role in bone diseases such as OA and osteoporosis (OP) [27]. After the initiation of autophagy, the ULK1/LC3 axis runs through the formation, prolongation, and maturation of APs. At the same time, p62 binds to LC3 II, mediates the binding of substrate to APs, and is then degraded and recovered by lysosomes [28,29]. Therefore, the activation of LC3 II and concomitant decrease in p62 together signify an increase in autophagy. Previous studies have shown that the mitochondria and apoptosis change during the pathogenesis of KOA due to the decrease in autophagy [30]. In our study, osthole treatment increased the level of LC3 II and decreased the p62 level, which was accompanied by an increase in the number of Aps by TEM in vitro and in vivo. Additionally, the enhancement of autophagy improved cartilage matrix degeneration and apoptosis, while the autophagy inhibitor 3-MA reversed this trend. 3-MA, a classical inhibitor of class III phosphatidylinositol 3-kinase (PI3K), is characterized by its ability to inhibit the formation of APs, and is thus widely used in autophagy studies in various cells such as chondrocytes [31], nucleus pulposus derived stem cells [32], and multiple myeloma cells [33]. The study of these cells also showed no significant effect of 3-MA on life processes such as apoptosis. Therefore, the effect of 3-MA on apoptosis was not tested separately in the present study. These findings indicate that osthole altered KOA progression by activating autophagy.

Autophagy has been reported to affect the development of KOA, and the AMPK pathway is the core regulator of autophagy [34,35]. ULK1, as a booster of autophagy, plays a significant role in the formation of APs, and its activity is influenced by phosphorylation. Furthermore, AMPK is considered to be an upstream regulatory kinase of ULK1, which can phosphorylate Ser317 and Ser377 on ULK1 to activate autophagy [36,37]. Previous studies have reported that phosphorylated AMPK promotes autophagy and inhibits apoptosis of chondrocytes, thereby playing a protective role against KOA, which is consistent with our findings [38,39]. In the present study, we confirmed that osthole upregulates AMPK phosphorylation and that AMPK promotes autophagy by phosphorylating ULK1. Additionally, we found that autophagy was activated and that apoptosis was inhibited strongly in IL-1β-cocultured chondrocytes at 24 h after osthole treatment. Osthole-mediated phosphorylation of ULK1, activation of autophagy, and inhibition of apoptosis in these chondrocytes was reversed by treatment with the AMPK inhibitor Dorsomorphin. In vivo, high-dose osthole also promoted the phosphorylation of AMPK and ULK1 in the knee cartilage of KOA rats. The AMPK/ULK1 pathway was implicated in osthole-related autophagy promotion in these findings.

Notably, the minimum effective dose and the maximum dose of osthole were predicted by testing the viability of chondrocytes treated with osthole in vitro. The results showed that the range of both was not large, so we believe that in the future, if osthole is used in clinical treatment, its dose should be strictly controlled. In addition, modification of osthole to optimize its dose range is also a feasible strategy.

Our study has a few limitations that should be acknowledged. Naturally, there is the ever-present question of translatability to human subjects when using any animal model. Moreover, micro-computed tomography and high-resolution image analysis were not used to quantify the effect of osthole on KOA. We intend to probe further into the latter topic in future studies.

## 4. Materials and Methods

### 4.1. Reagents and Antibodies

Osthole (M10391-01), 3-MA (M2296-06), and Dorsomorphin (M2238-04) were purchased from AbMole (Houston, Texas, USA). Dulbecco’s modified Eagle’s medium (DMEM, ED-DGP01), phosphate buffer saline (PBS, ES4011), fetal bovine serum (FBS, ES-FBS001), penicillin/streptomycin (ES0015), 0.25% trypsin (ES0014), and Cell Counting Kit-8 (CCK-8, ES7011) were obtained from YiShan Biotechnology Co., Ltd. (Shanghai, China). Toluidine Blue (G3660) was purchased from Solarbio Science & Technology Co., Ltd. (Beijing, China). The Annexin V-FITC/PI Apoptosis Detection Kit (A211-02) was provided by Vazyme Biotechnology Co., Ltd. (Nanjing, China). Enzyme-linked immunosorbent assay (ELISA) kit for rat 1L-1β (70-EK301B/3-96) and ELISA kit for rat TNF-α (70-EK382/3-96) were supplied by Multi sciences Technology Co., Ltd. (Hangzhou, China). The recombinant rat IL-1β (400-01B-10UG) was obtained from PeproTech (Rocky Hill, NJ, USA). The dimethyl sulfoxide (DMSO, ST038) was provided by Beyotime (Shanghai, China). Anti-p62 antibody (#5114), anti-LC3A/B antibody (#12741), anti-AMPKa antibody (#5831), anti-p-AMPK antibody (#2535), anti-ULK1 antibody (#8054), and anti-p-ULK1 antibody (#14202) were provided from Cell Signaling Technology (Danvers, MA, USA). Anti-collagen type II α1 (COL2A1, 28459-1-AP) antibody, anti-MMP13 antibody (18165-1-AP), and anti-GAPDH antibody (10494-1-AP) were purchased from Proteintech (Rosemont, IL, USA). Anti-ADAMTS5 antibody (A2836) was purchased from ABclonal (Wuhan, China).

### 4.2. Culture and Identification of Primary Chondrocytes from SD Rats

The hyaline cartilage of the knee joint of Sprague Dawley rats (male, 1-week-old) was minced, and then digested with 0.25% trypsin and 0.2% collagenase II at 37 °C for 30 min and 4 h, respectively. Incubation of the chondrocytes with 5% CO_2_ at 37 °C was performed in DMEM containing 10% FBS and 1% penicillin/streptomycin. Upon reaching 80–90% confluence, chondrocytes were subcultured at a 1:3 ratio. After being identified using toluidine blue and anti-COL2A1 (1:200) antibody staining, second-passage chondrocytes were used for the experiments in this report.

### 4.3. Cell Treatment

Recombinant rat IL-1β (10 ng/mL) was prepared in sterile distilled H2O and diluent to simulate OA. Osthole was directly prepared from DMSO and diluted in complete medium to 0.1% DMSO before use. Chondrocytes were divided into six treatment groups: (1) control [untreated]; (2) IL-1β; (3) DMSO [complete medium + 0.1% DMSO]; (4) osthole [osthole (100 µM) suspended in complete medium + 0.1% DMSO]; (5) osthole + 3-MA (autophagy inhibitor) [osthole (100 µM) and 3-MA (10 µM) [40] suspended in complete medium + 0.1% DMSO]; (6) osthole + Dorsomorphin (AMPK inhibitor) [osthole (100 µM) and Dorsomorphin (10 µM) suspended in complete medium + 0.1% DMSO]. We cultured all groups except the control for 24 h with 10 ng/mL IL-1β [41].

### 4.4. Cell Viability Assay

The maximally effective safe dose of osthole was determined using the CCK-8 assay. Chondrocytes were transferred to 96-well plates and incubated at a concentration of 5000 cells/well. Plated cells were then treated with osthole dissolved in a culture medium at different concentrations (0, 10, 50, 100, 150, 200, 400, or 600 µM) for 24 h to investigate the safe dose by CCK-8. Normal culture medium was used in the blank group, and 10 ng/mL IL-1β was used in the drug group. After 24 h, the medium was discarded. Then, normal medium (100 µL) was added to the blank group, and osthole medium at different concentrations (0, 25, 50, 75, 100, 125, 150, and 200 µM) was added to the drug group, respectively. After 24 h incubation, we estimated the cell viability with the absorbance values of CCK-8 reactions at 450 nm.

### 4.5. Apoptosis Detection

Second passage cells were obtained following treatment, as described in Section 2.2, and stained with propidium iodide (PI) and fluorescein isothiocyanate-conjugated Annexin V according to the manufacturer’s instructions. Cell apoptosis was determined by flow cytometry (C6 Plus; BD, USA).

### 4.6. Transmission Electron Microscopy (TEM)

Second passage chondrocytes were fixed in 2.5% glutaraldehyde for 12 h after treatment and then in 1% osmium tetroxide for 2 h. Following gradient dehydration, the cells were embedded in epoxy resin and cut into 70 nm ultrathin sections. Similarly, cartilage tissues of rats were rapidly cut into 2 mm^3^ pieces and immersed in a fixative solution for 2 h. Finally, the sections were observed under a transmission electron microscope (TEM) (Hitachi, Tokyo, Japan) to calculate the number of autophagosomes.

### 4.7. Immunofluorescence Staining

Expression of autophagy-related markers (LC3A/B) and 14hosphor-AMPKα was analyzed by immunofluorescence. Briefly, chondrocytes on slides were fixed using 4% paraformaldehyde and permeabilized using 0.1% Triton X-100 in PBS. Then, the cells were incubated with primary antibodies against LC3A/B (1:200) and 14hosphor-AMPKα (1:200) at 4 °C overnight. The next day, the appropriate secondary antibody was added and incubated at room temperature in the dark for 90 min. After washing with PBS, chondrocytes were incubated with DAPI at room temperature. The slides were photographed by a fluorescence microscope (Nikon, Tokyo, Japan).

### 4.8. Western Blotting

Protein was collected from chondrocytes using Radio Immunoprecipitation Assay (RIPA) (G2002; Servicebio, Wuhan, China) and quantified with a Bicinchoninic acid (BCA) assay kit (PC0020; Solarbio). Samples (50 µg/lane) were separated by Sodium Dodecyl Sulfate Polyacrylamide Gel Electrophoresis (SDS-PAGE), transferred onto polyvinylidene difluoride (PVDF) membranes, and incubated with primary antibodies against COL2A1 (1:1000), MMP13 (1:1000), ADAMTS5 (1:1000), LC3A/B (1:500), p62 (1:1000), 15hosphor-AMPKα (1:500), AMPKα (1:1000), 15hosphor-ULK1 (1:500), ULK1 (1:1000), and GAPDH (1:5000), followed by peroxidase-conjugated goat anti-rabbit secondary antibody (1:5000). The bands were visualized using an enhanced chemiluminescence (ECL) kit (E412-02; Vazyme). The ratio of the absorbance of target band to that of GAPDH band was used to calculate the expression level of the proteins.

### 4.9. Quantitative Real-Time Polymerase Chain Reaction Analysis (qPCR)

Total RNA was extracted from chondrocytes using TRIzol reagent (R401-01; Vazyme) and amplified to cDNA by HiScript II Q RT reagent kit (R223-01; Vazyme). Quantitative PCR (qPCR) analysis was performed using ChamQ SYBR qPCR Master Mix (R311-02; Vazyme) with CFX96 Touch (Bio-Rad, Hercules, CA, USA). Fold differences in relative mRNA expression were processed using the 2^−ΔΔCt^ method, with GAPDH used as the internal control. The primers are presented in Table 1.

### 4.10. Animals

Forty male Sprague Dawley rats (3-week-old, 100–120 g) were purchased from Beijing Vital River Laboratory Animal Technology Co., Ltd. [certificate: SCXK(JING)2021-0006] and housed under specific pathogen free (SPF) conditions in our facility. All animal procedures were approved by the Institutional Animal Care and Use Committee of the Affiliated Hospital of Shandong University of Traditional Chinese Medicine (AWE-2019-043) and conducted in accordance with the United States National Institutes of Health guide for the care and use of Laboratory animals. After 1 week of adaptive feeding, the rats were randomly assigned into five groups: control group (gavage equivalent volumes of normal saline once a day; *n* = 8); model group (gavage equivalent volumes of normal saline once a day; *n* = 8); low- (50 mg/kg/day; gavage once a day; *n* = 8), mid- (75 mg/kg/day; gavage once a day; *n* = 8), and high-dose osthole groups (100 mg/kg/day; gavage once a day; *n* = 8) [42,43]. With the exception of the control group, rats were subjected to the modified Hulth method [11] to establish experimental KOA. Briefly, the left knee anterior cruciate and medial collateral ligaments were transected, and the medial meniscus was resected. After 4 weeks of intervention, all fasted rats were euthanatized with 1% pentobarbital (100 mg/kg). In the end, articular cartilages and serum of peripheral blood were harvested for subsequent experiments.

### 4.11. Histology and IHC Analysis

Samples of articular cartilage were fixed with 4% paraformaldehyde, decalcified in 10% ethylenediaminetetraacetic acid disodium salt (EDTA-2Na) solution, dehydrated in ethanol, embedded in paraffin, and then sliced into 5 µm sections. After dewaxing and rehydration, the sections were stained with hematoxylin and eosin and safranin O/fast green in accordance with the manufacturer’s instructions. The sections used for IHC were probed with primary antibodies against COL2A1 (1:200), MMP13 (1:200), LC3A/B (1:500), p62 (1:500), 16hosphor-AMPKα (1:200), and 16hosphor-ULK1 (1:200). Subsequently, the sections were incubated with a secondary antibody (1:1000) and stained with a diaminobenzidine (DAB) substrate and counterstained with hematoxylin. The cartilage histopathological features were quantified with Mankin scores [44] by two investigators in a blinded manner.

### 4.12. Enzyme-Linked Immunosorbent Assay (ELISA)

After centrifuging the peripheral blood obtained from the rats, the sera (supernatant) were analyzed by conventional ELISA kits according to the manufacturer’s instructions, and finally OD values at 450 nm wavelength were detected using a microplate reader to measure the levels of the proinflammatory cytokines IL-1βand TNF-α.

### 4.13. Statistical Analysis

GraphPad Prism (v.8.0; GraphPad Software, San Diego, CA, USA) was used for statistical analysis. All quantitative data are expressed as the mean ± standard deviation (X ± S). One-way analysis of variance (ANOVA) was used to determine the significance of differences between groups and Brown–Forsythe and Welch ANOVA were used when the variance was unequal. A value of *p* < 0.05 was considered statistically significant.

## 5. Conclusions

Using IL-1β-treated rat chondrocytes, we demonstrated that osthole delayed OA degeneration in vitro and that administration of osthole by oral gavage protected joint cartilage in a KOA rat model. Furthermore, osthole downregulated apoptosis and cartilage matrix degeneration in vitro and in vivo, likely by activating autophagy through phosphorylation of AMPK/ULK1. We conclude that osthole may be a potential therapeutic drug for the treatment of patients with KOA.

## Figures and Tables

**Table 1:** Sequences of the primers used for qPCR.

Name	Primer	Sequence
COL2A1	Forward Reverse	GACGCCACGCTCAGTC TCTCCGCTCTTCCACTCTG
ADAMTS5	Forward Reverse	GCATTACCTGCTGACCCT TTCTTGCTCACCTCCAGAC
MMP13	Forward Reverse	TGGGCCTTCTGGTCTTC GTTGTAGCCTTTGGAGATG
LC3A/B	Forward Reverse	TGCACTCGCCTTGTACG CTCTTCCGTTGCTGTTGC
P62	Forward Reverse	GACTTGGTCGCCTTCTCC ATGCTTCGTGCCTCCTG
GAPDH	Forward Reverse	CCTTCCGTGTCCCCACT GCCTGCTTCACCACCTTC

## Data Availability

All datasets generated in this study are included in the article.

## Sample Availability

Samples of the compounds are not available from the authors.
